# 18F-FDG PET-CT for Evaluation of Cardiac Angiosarcoma: A Case Report and Review of Literature

**DOI:** 10.4274/mirt.02486

**Published:** 2015-02-15

**Authors:** Varun Singh Dhull, Punit Sharma, Anirban Mukherjee, Manisha Jana, Chandrasekhar Bal, Rakesh Kumar

**Affiliations:** 1 All India Institute of Medical Sciences, Department of Nuclear Medicine, New Delhi, India; 2 All India Institute of Medical Sciences, Department of Radiodiagnosis, New Delhi, India

**Keywords:** Hemangiosarcoma, Heart, PET Scan, 18F-FDG, Bone, metastasis

## Abstract

Cardiac angiosarcomas are rare neoplasms. We here present the case of a 24 year old male with a cardiac mass which was characterised as malignant on 18F-Fluorodeoxyglucose (18F-FDG) positron emission tomography-computed tomography (PET-CT). In addition 18F-FDG PET-CT also demonstrated pericardial infiltration and bone metastases. The tumor was confirmed to be angiosarcoma on biopsy and palliative chemotherapy was started. Here we have highlighted the potential role of 18F-FDG PET-CT in patients with cardiac angiosarcoma and presented a brief review.

## Abstract (tr)

Kardiyak anjiyosarkomlar nadir neoplazilerdir. Bu makalede 18F-Florodeoksiglukoz (18F-FDG) pozitron emisyon tomografi-bilgisayarlı tomografi (PET-BT) ile malin olduğu saptanan kardiyak kitlesi olan 24 yaşında bir olguyu sunduk. 18F-FDG PET-BT aynı zamanda perikardiyal infiltrasyon ve kemik metastazını da gösterdi. Tümör, biyopsi sonucunda anjiyosarkom olarak doğrulandı ve palyatif kemoterapiye başlandı. Bu makalede kardiyak anjiyosarkomlu hastalarda 18F-FDG PET-BT’nin potansiyel rolünü vurguladık ve kısa bir derleme sunduk.

## INTRODUCTION

Primary cardiac tumors are rare, with an incidence ranging from 0.001% to 0.028% in autopsy reports. About one fourth of these tumors are malignant, with angiosarcoma being the most common malignant cardiac tumor (1,2). Cardiac angiosarcomas are neoplasms of mesenchymal cells. Only 200 cases have been described in the literature (3). These tumors are resistant to radiation and chemotherapeutic agents, therefore, surgical resection remains the treatment of choice. But the diagnosis is often delayed as the symptoms are usually non-specific. The tumor has a high mortality rate as it has a tendency for local relapse along with a high incidence of systemic metastases. Survival may range from a few days to years (4,5). As cardiac angiosarcomas are highly malignant tumors with poor prognosis, early diagnosis is mandatory. Therefore, it is important to determine the exact extent of the primary lesion, to detect local recurrence and distant metastases for appropriate therapy management (6). Here, we have aimed to report the potential benefits of 18F-Fluorodeoxyglucose (18F-FDG) positron emission tomography-computed tomography (PET-CT) imaging for examination of malignant potential and exploration of distant metastases in a young patient of cardiac angiosarcoma. In addition we have reviewed the published literature regarding the utility of 18F-FDG PET-CT in cardiac angiosarcoma.

## CASE REPORT

**Figure 1:**
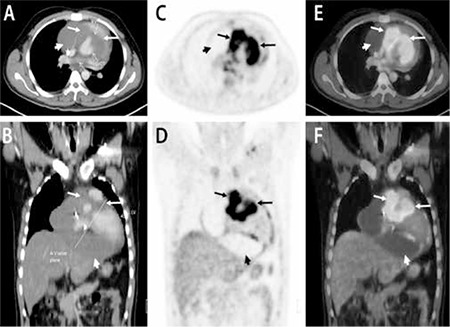
Contrast enhanced CT (Transaxial A, Coronal B) revealed a large ill defined non enhancing soft tissue density mass (bold arrows) in the interventricular septum (proximal two-third) extending into the right ventricle upto right ventricle outflow tract, the aortic root and the right A-V valve with a small extension along the left ventricular wall. 18F-FDG PET (Transaxial C, Coronal D) and contrast enhanced 18F-FDG PET-CT (Transaxial E, Coronal F) shows increased 18F-FDG uptake in the soft tissue mass seen on contrast enhanced CT (arrows, SUVmax-8.3). Also noted is loculated pericardial effusion without significant 18F-FDG uptake on 18F-FDG PET (arrowheads).RV-right ventricle; LV-left ventricle; RVOT-RV outflow tract; LVOT-LV outflow tract; A-V-atrio-ventricular.

**Figure 2:**
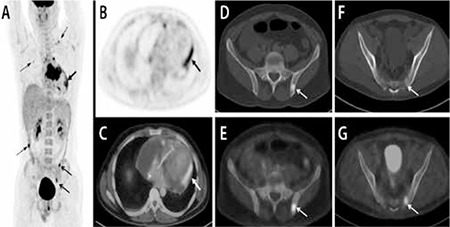
Apart from the primary tumor, PET Maximum Intensity Projection (MIP) image shows increased 18F-FDG uptake along the left lateral border of the heart (A, bold arrow) along with two foci of increased 18F-FDG uptake in left hemipelvis (arrows). Other foci of benign FDG uptake (broken arrows) on MIP image were seen in left supraclavicular region (central venous line), right hemithorax (right lung infection) and ascending colon (normal colonoscopy).Transaxial 18F-FDG PET (B) and contrast enhanced 18F-FDG PET-CT (C) show moderate pericardial effusion with increased 18F-FDG uptake in pericardium (arrow) likely infiltration (SUVmax-5.1). Minimal right pleural effusion is also noted. Transaxial CT (D) and 18F-FDG PET-CT (E) show a sclerotic lesion in left iliac bone (arrow) with increased 18F-FDG uptake (SUVmax-4.5). Transaxial CT (F) and 18F-FDG PET-CT (G) show another focus of increased 18F-FDG uptake in left ala of sacrum (SUVmax-3.7) with minimal sclerosis on CT (arrow).

A 24 year old male patient was admitted to the emergency department with progressive dyspnoea. Patient had tachycardia (PR: 100/minute) and low blood pressure (100/60 mmHg) at presentation. The intensity of the heart sounds was reduced. All other physical examinations were normal. On chest X-ray a massive cardiomegaly was seen. Transthoracic echocardiography showed a large hypoechoic ill-defined cardiac mass. Subsequently patient underwent contrast enhanced CT that showed a large mass draping the cardiac root and ascending aorta, left ventricular outflow tract, right ventricular outflow tract, infundibulum and the basal interventricular septum (Figure 1a, 1b). Patient was then referred to our department for 18F-FDG PET-CT for further characterisation of the cardiac mass as well as for metastatic work up. The patient fasted overnight. Blood glucose level was 96 mg/dl. A dose of 370 MBq of 18F-FDG was injected intravenously. PET-CT acquisition was done after a 45 minute uptake period. 50 ml of non ionic iodinated intravenous contrast (Visipaque, 320 mg I/ml, GE) was administered and scan was acquired after a delay of 50 seconds. Contrast enhanced 8F-FDG PET-CT revealed a large ill defined non enhancing soft tissue density mass (single largest dimension 8.1 cm) in the interventricular septum (proximal two-third) extending into the right ventricle upto right ventricle outflow tract, the aortic root and the right A-V valve with a small extension along the left ventricular wall with increased 18F-FDG uptake (SUVmax-8.3) (Figure 1c-1f). Pericardial and minimal right pleural effusion were noted. Pericardial effusion was loculated mainly on the right side and the inferior surface of the heart (Figure 2a). Also, increased 18F-FDG uptake was seen in the pericardium, likely due to pericardial infiltration by the tumor (SUVmax-5.1) (Figure 2b, 2c). The SUVmax of the normal myocardium was 1.5. Apart from the cardiac findings, there was a sclerotic lesion in left iliac bone with increased 18F-FDG uptake (SUVmax-4.5) (Figure 2d-2e). Another focus of increased 18F-FDG uptake was noted in left ala of sacrum (SUVmax-3.7) with minimal sclerosis on CT (Figure 2f-2g). 18F-FDG PET-CT findings were suspicious for a cardiac tumor with skeletal metastases. Biopsy from the cardiac lesion was performed which showed spindle cell neoplasm with CD-31 positivity, suggesting angiosarcoma. Hence, the diagnosis of cardiac angiosarcoma with skeletal metastases was reached. Patient is undergoing multiagent chemotherapy and has shown significant clinical improvement at 5 months of follow up.

**Literature Review and Discussion**

Early diagnosis and prompt management is crucial for the survival of the patients with cardiac angiosarcoma. With the availability of various non-invasive and advanced diagnostic tools, an early diagnosis of this rare lesion is possible (7). Although a positive correlation has been found between 18F-FDG accumulation and the degree of malignancy for many tumors, high 18F-FDG uptake in myocardium does not necessarily mean a malignant lesion. The degree and extent of 18F-FDG myocardial activity may be heterogeneous and variable. Patients with myocardial ischemia, coronary artery disease, atherosclerotic plaques, etc may have a focal increased 18F-FDG uptake (8,9). Moreover, respiratory motion can sometimes lead to inhomogeneties in myocardial FDG uptake, more so in the lateral and anterior regions and to a lesser extent in the septal region (10).

18F-FDG PET-CT has been evaluated for characterisation, staging and restaging of cardiac angiosarcomas. A brief review of literature in this regard is presented in Table (11,12,13,14,15,16,17,18,19,20,21,22,23). Rahbar et al. in their study on 24 patients with cardiac tumors (including 6 angiosarcomas) concluded that with a cut-off SUVmax of 3.5, 18F-FDG PET-CT could be used to noninvasively determine malignant tumors with a sensitivity of 100% (22). In the present case too, the 18F-FDG uptake was high in the cardiac lesion (SUVmax-8.3), which pointed towards the malignant nature which was confirmed on biopsy.

18F-FDG PET-CT is widely employed for staging of various tumors. Being a whole body imaging modality, 18F-FDG PET-CT is useful for the detection of distant metastases which may be missed on routine conventional imaging modalities. 18F-FDG PET-CT has been sparsely used for staging of cardiac angiosarcoma, given the rarity of such tumors (Table 1). In such patients 18F-FDG PET-CT can accurately determine the extent of the primary tumour as well as demonstrate distant metastases, if present. In the present case too, 18F-FDG PET-CT clearly demonstrated the extent of the primary tumor. In addition the patient had skeletal metastases which were detected on 18F-FDG PET-CT. In early stages without distant metastases, surgery followed by postoperative chemotherapy remains the treatment of choice while palliative chemotherapy with/without cytoreductive surgery is the pathway of management in patients with metastatic disease (11,24). As our patient had metastatic disease on PET-CT, he is undergoing chemotherapy and showed symptomatic improvement. It is to be remembered that the prognosis of metastatic cardiac angiosarcoma remains poor even with chemotherapy and thus 18F-FDG PET-CT can categorise such patients into prognostic groups.

Thus, the present case and the published literature show the potential of 18F-FDG PET-CT in management of patients with cardiac angiosarcoma starting from helping in reaching a diagnosis to staging to restaging and possibly prognostication. However, false positive causes should be kept in mind.

## Figures and Tables

**Table 1:**
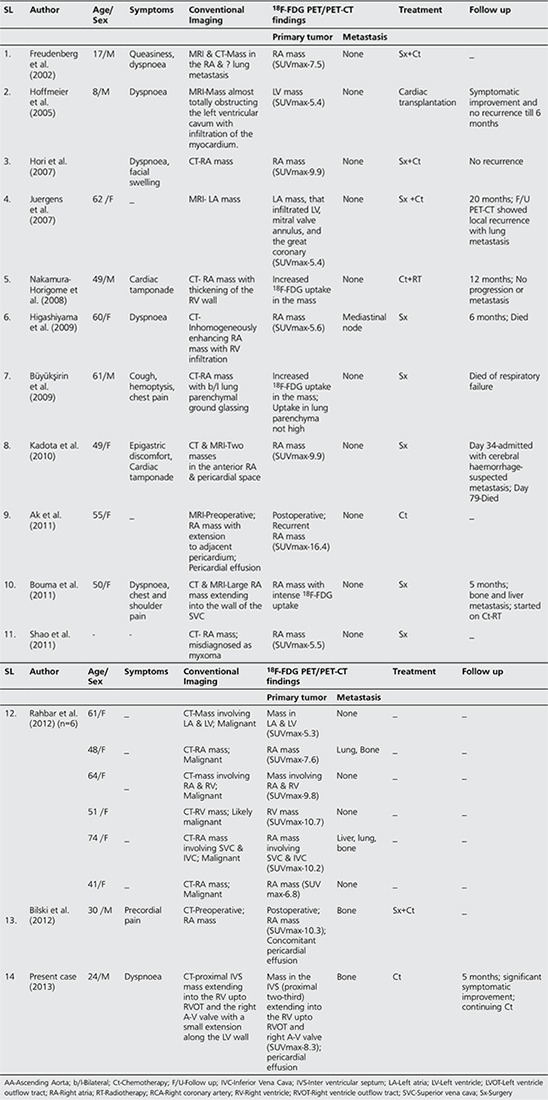
Review of literature-18F-FDG PET/PET-CT for primary cardiac angiosarcoma reported in the English literature

## References

[1] Burke A, Virmani R., Rosa J, Sobin LH (1996). Tumors of the heart and great vessels. Atlas of tumor pathology.

[2] Bussani R, De-Giorgio F, Abbate A, Silvestri F (2007). Cardiac metastases. J Clin Pathol.

[3] Matheis G, Beyersdorf F (1995). Primary cardiac angiosarcoma. A case report.. Cardiology.

[4] Herrmann MA, Shankerman RA, Edwards WD, Shub C, Schaff HV (1992). Primary cardiac angiosarcoma: a clinicopathologic study of six cases. J Thorac Cardiovasc Surg.

[5] Nakamichi T, Fukuda T, Suzuki T, Kaneko T, Morikawa Y (1997). Primary cardiac angiosarcoma: 53 months survival after multidisciplinary therapy. Ann Thorac Surg.

[6] Tokmak E, Özkan E, Yaga S, Kir KM (2011). F18-FDG PET/CT Scanning in Angiosarcoma: Report of Two Cases. Mol Imaging Radionucl Ther.

[7] Butany J, Yu W (2000). Cardiac angiosarcoma: two cases and a review of the literature. Can J Cardiol.

[8] Minamimoto R, Morooka M, Miyata Y, Ito K, Okasaki M, Hara H, Okazaki O, Moroi M, Kubota K (2013). Incidental focal FDG uptake in heart is a lighthouse for considering cardiac screening. Ann Nucl Med.

[9] Hanif MZ, Ghesani M, Shah AA, Kasai T (2004). F-18 fluorodeoxyglucose uptake in atherosclerotic plaque in the mediastinum mimicking malignancy: another potential for error. Clin Nuc Med.

[10] Le Meunier L, Maass-Moreno R, Carrasquillo JA, Dieckmann W, Bacharach SL (2006). PET/CT imaging: effect on respiratory motion on apparent myocardial uptake. J Nucl Cardiol.

[11] Freudenberg LS, Rosenbaum SJ, Schulte-Herbruggen J, Eising EG, Lauenstein T, Wolff A, Bockisch A (2002). Diagnosis of a cardiac angiosarcoma by fluorine-18 fluordeoxyglucose positron emission tomography. Eur Radiol.

[12] Hoffmeier A, Etz C, Schmid C, Debus V, Kehl HG, Ozgun M (2005). Cardiac Transplantation for Giant Sarcoma of the Left Ventricle. Circulation.

[13] Hori Y, Funabashi N, Miyauchi H, Nakagawa K, Shimura H, Miyazaki M, Kozono H, Nagai Y, Ishikura H, Nagai T, Kobayashi Y, Komuro I (2007). Angiosarcoma in the right atria demonstrated by fusion images of multislice computed tomography and positron emission tomography using F-18 Fluoro-Deoxyglucose. Int J Cardiol.

[14] Juergens KU, Hoffmeier A, Riemann B, Maintz D (2007). Early detection of local tumour recurrence and pulmonary metastasis in cardiac angiosarcoma with PET-CT and MRI. Eur Heart J.

[15] Nakamura-Horigome M, Koyama J, Eizawa T, Kasai H, Kumazaki S, Tsutsui H, Koiwai K, Oguchi K, Kinoshita O, Ikeda U (2008). Successful treatment of primary cardiac angiosarcoma with docetaxel and radiotherapy. Angiology.

[16] Higashiyama S, Kawabe J, Hayashi T, Kurooka H, Oe A, Kawamura E, Shiomi S (2009). Effectiveness of preoperative PET examination of huge angiosarcoma of the heart. Clin Nucl Med.

[17] Büyükşirin M, Yakut N, Polat G, Usalan AK, Gökdoğan T, Yücel N, Kupeli A (2012). Primary cardiac angiosarcoma and diffuse pulmonary hemorrhage: a case report. Turk Gogus Kalp Damar.

[18] Kadota S, Matsuda M, Umei N, Izuhara M, Baba O, Mitsuoka H (2010). A case of right atrial angiosarcoma: The utility of PET and CT fusion imaging in detecting a malignant cardiac tumor. Journal of Cardiology Cases.

[19] Ak I, Ciftçi OD, Ustünel Z, Sivrikoz MC (2011). Atrial angiosarcoma imaged by F-18 FDG PET/CT. Anadolu Kardiyol Derg.

[20] Bouma W, Lexis CPH, Willems TP, Suurmeijer AJH, Ebels T, Mariani MA (2011). Successful surgical excision of primary right atrial Angiosarcoma. Journal of Cardiothoracic Surgery.

[21] Shao D, Wang SX, Liang CH, Gao Q (2011). Differentiation of malignant from benign heart and pericardial lesions using positron emission tomography and computed tomography. Journal of Nuclear Cardiology.

[22] Shao D, Wang SX, Liang CH, Gao Q (2011). Differentiation of malignant from benign heart and pericardial lesions using positron emission tomography and computed tomography. Journal of Nuclear Cardiology.

[23] Bilski M, Kaminski G, Dziuk M (2012). Metabolic activity assessment of cardiac angiosarcoma by 18FDG PET-CT. Nucl Med Rev Cent East Eur.

[24] Baay P, Karwande SV, Kushner JP, Olsen S, Renlund DG (1994). Successful treatment of a cardiac angiosarcoma with combined modality therapy. J Heart Lung Transplant.

